# Targeting the angiotensin II type 2 receptor (AT2R) in colorectal liver metastases

**DOI:** 10.1186/1475-2867-10-19

**Published:** 2010-06-28

**Authors:** Eleanor I Ager, Way W Chong, Shu-wen Wen, Christopher Christophi

**Affiliations:** 1 Department of Surgery, The University of Melbourne, Austin Health, Heidelberg, Victoria, Australia

## Abstract

**Background:**

Blockade of the angiotensin (ANG) II type 1 receptor (AT1R) inhibits tumour growth in several cancers, including colorectal cancer (CRC) liver metastases. While AT1R blockade has been extensively studied, the potential of targeting the antagonistically acting AT2R in cancer has not been investigated. This study examined the effect of AT2R activation with the agonist CGP42112A in a mouse model of CRC liver metastases.

**Results:**

*In vitro*, mouse CRC cell (MoCR) proliferation was inhibited by treatment with CGP42112A in a dose dependent manner while apoptosis was increased. Immunofluorescent staining for key signalling and secondary messengers, PLA2 and iNOS, were also increased by CGP42112A treatment *in vitro*. Immunohistochemical staining for proliferation (PCNA) and the apoptosis (active caspase 3) markers confirmed a CGP42112A-associated inhibition of proliferation and induction of apoptosis of mouse CRC cells (MoCR) *in vivo*. However, angiogenesis and vascular endothelial growth factor (VEGF) appeared to be increased by CGP42112A treatment *in vivo*. This increase in VEGF secretion by MoCRs was confirmed *in vitro*. Despite this apparent pro-angiogenic effect, a syngenic orthotopic mouse model of CRC liver metastases showed a reduction in liver to body weight ratio, an indication of tumour burden, following CGP42112A treatment compared to untreated controls.

**Conclusions:**

These results suggest that AT2R activation might provide a novel target to inhibit tumour growth. Its potential to stimulate angiogenesis could be compensated by combination with anti-angiogenic agents.

## Background

Metastasis to the liver is the leading cause of death in patients with colorectal cancer (CRC)[1]. For the majority of these patients the only treatment option is palliative chemotherapy [2,3]. The renin angiotensin system (RAS) is expressed in several cancers and regulates proliferation and angiogenesis in several pathological conditions [4,5]. Experimental animal models show a stimulatory effect of the key RAS peptide angiotensin (ANG) II through the ANG II type 1 receptor (AT1R) on tumour growth, while blockade this pathway inhibits tumour growth[6,7], including in a mouse model of CRC liver metastases [8]. However, the effects of the RAS can also be mediated through an alterative receptor, the angiotensin II type 2 receptor (AT2R), as well as an alternative peptide ANG-(1-7) and its receptor (the MasR). The AT2R generally exerts actions antagonistic to the AT1R including inhibition of proliferation and angiogenesis [9,10] and promotion of apoptosis [11] and while AT1R blockade has been extensively studied in the context of cancer treatment, the potential of targeting the AT2R in cancer has not been investigated. AT2R expression has been documented in blood vessels of human pituitary adenomas[12] and both the AT1R and AT2R stimulate vascular endothelial growth factor (VEGF) secretion by rat pituitary tumour cells [13]. AT2R activation has, however, also been shown to inhibit VEGF signalling [9] and angiogenesis [10], suggesting it can mediate both pro- and anti-angiogenic actions. Here we targeted the AT2R via the agonist, CGP42112A[14,15], in an orthotopic syngenic mouse model of CRC liver metastases in which we previously demonstrated inhibition of tumour growth following AT1R blockade [8].

## Methods

### In vivo model and cell lines

The mouse colorectal cancer (MoCR) cell line used in both the *in vitro *and *in vivo *experiments was harvested from a dimethylhydrazine- induced colon carcinoma in a CBA mouse at a stage known to metastasise to the liver [16]. *In vitro*, MoCR cells were maintained in RPMI/5%FBS in an environment of 5%CO_2_/95% air at 37°C. Sub-confluent cultures were used at passages 4 to 15. Liver metastases were induced as described previously [8,16]. Briefly, 25000 MoCR cells were injected into the spleen of 6 to 8 week old male CBA mice and, after 3 minutes, the spleen removed to confine metastases to the liver. All experiments were approved by the Austin Health Animal Ethics Committee. Liver, kidney, and lung samples were collected and fixed in fresh 4% PFA.

### Drugs/agents and treatments

The AT2R agonist CGP42112A (Sigma-Aldrich, C160) at 0.6 μg/kg/hr (solubilised in physiological saline) was given via osmotic mini pump (Alzet^® ^osmotic pumps 1004) implanted at the time of tumour induction [14,17]. Control animals received no treatment. Treatments continued from the time of tumour induction to tissue collection at day 21. *In vitro *studies also used CGP42112A at concentrations of 0.1 μM or 1 μM solubilised in negative control medium (RPMI/0.1% FBS) or positive control (RPMI/2%FBS).

### CFSE and PI staining

To provide a direct measure of cell proliferation and apoptosis a combination of CFSE (Cell Trace CFSE cell proliferation kit, Invitrogen, #C34554) and PI (propidium iodide) were used in a FACs analysis of cells treated for 24, 48, 72, or 120 hours. Between 4 and 8 wells across at least 4 plates were used in analyses. Cells were stained with CFSE as per manufacturer's instructions and allowed to attach overnight in a 6 well plate at a density of 1 to 2 × 10^5 ^cells/well. After attachment, cells were treated for the request time with 0.1 μM or 1 μM CGP42112A in a background of either 0.1% FBS/RPMI (negative growth control) or 2% FBS/RPMI (positive growth control). At terminal time points the cells were washed with PBS and lifted before centrifuging, washing, and suspending in PBS. PI was added within 5 minutes of FACs analysis. Data was analysed using Weasel (V2).

### Immunohistochemistry and immunocytochemistry

Proliferation (PCNA; rabbit polyclonal, Santa Cruz *sc-7907*), apoptosis (active caspase 3; rabbit polyclonal, R&D Systems *AF835*), angiogenesis (CD34 neovascularisation marker; rat anti-mouse, Abd Serotec MCA18256), and VEGF (CalBiochem, PC315) were assessed in PFA-fixed paraffin embedded tissues. PCNA was used at the concentration of 0.143 μg/ml, active caspase-3 at 1.0 μg/ml, CD34 at 0.1 μg/ml, and VEGF at 1.5 μg/ml. Non-immunized rabbit IgG (Santa Cruz, sc-2027), at 1.0 μg/ml, was used as a negative control. Endogenous peroxidases were blocked with 3% H_2_O_2 _and non-specific binding inhibited with 10% normal goat serum (Zymed, 01-6201). Slides were incubated with primary antibodies at 37°C for 1 hour and then 4°C overnight. Slides were then incubated with the secondary antibody (Dako Envision^+ ^Goat anti-rabbit HRP secondary *4011*) for PCNA, caspase 3 and VEGF, and the Rat on Mouse AP-polymer kit (Biocare Medical; RT518H) for CD34 for 1 hour at 37°C before visualisation with DAB or, for CD34, Vulcan fast red (Applied Medical FR805H). Slides were counterstained with Mayer's haematoxylin. Cultured cells were grown on superfrost slides and stained for phospholypase A2 (PLA2; Abcam, ab58375) and inducible nitric oxide synthase (iNOS; Abcam, ab15323) after 24 hours of treatment with 0.1 μM and 1 μM CGP42112A or control. iNOS was used at a dilution of 1:50 and PLA2 at 1:100 over a 2 hour 37°C incubation before staining with the fluorescently-labelled secondary (Alexa Flour 594, A11012). UltraCruz Mounting medium (Santa Cruz Biotechnology, sc-24941, USA) containing 4',6-diamidino-2-phenylindole (DAPI), was used as a counter stain.

Images of stained tumours were taken using digital light microscope (Nikon Coolscope^®^, Nikon Corporation, Japan). For each tumour, 6-15 random images were taken at 40 × magnification and the number of PCNA or caspase 3 positive cells per area of viable tumour (excluding necrotic areas, stromal intrusions and any blood vessels or vascular lakes) determined using Image-Pro plus (version 5). CD34 was quantified as the percentage of positively stained endothelium per area of viable tumour (images taken at 10× magnification). For cultured cells grown on slides, aapproximately 20 random images were taken using the Leica DM4000 B Fluorescent microscope and associated software. Images were analysed using manual tag function under Image-Pro Plus and are expressed as the percent positive cells.

### Enzyme-linked immunosorbent assay (ELISA)

Conditioned media from MoCR cells (2.5 × 10^5 ^cells/ml in a background of 0% or 0.1% FBS/RPMI) was collected after treatment with CGP42112A (0.1 or 1 μM) for 24 hours. Two independent samples for each treatment condition were assessed in duplicate. The amount of VEGF protein secreted by treated and untreated control cells into culture medium was measured by ELISA (R&D Systems, Mouse VEGF165 Duoset, #DY493) following the manufacturer's instructions. Optical density of three repeat samples was measured with the Benchmark Plus microplate spectrophotometer (BioRad) at 450 nm absorbance and subtraction of 540 nm and Microplate Manager 5.2.1 software.

### Tumour burden

The wet liver and body weights were collected from all animals at the time of termination (day 21). The liver to body weight ratio was used as an indicator of tumour burden. The fixed livers were also transversely sliced into 1.5 mm sections with a multi blade fractionator and an image of liver sections taken using Lumenera Infinity4 digital CCD camera and tumour area (mm^2^) and the number of tumours per liver assessed using Image-Pro plus 6.0.

### Statistical analyses

Quantitative data are presented as mean ± SEM or boxplots showing the minimum value, first quartile, median, third quartile and maximum value. Statistical analyses were conducted using SPSS (Statistical Package for the Social Sciences, version 17, USA) or Microsoft Excel (2003). Normally distributed data were assessed by ANOVA. T-tests were used for comparisons between two means with Bonferroni adjustment for multiple comparisons. Games-Howell tests were used if groups had unequal variance. A probability (*P*) value of less than 0.05 was considered as statistically significant.

## Results

### CGP42112A inhibited MoCR proliferation in vitro

There was an initial delay in proliferation, probably as a result of the change in medium from 10% FBS to either 0.1% or 2%, at the 24 hours with the majority of cells undivided. However, after this point the average doubling time for MoCRs growing in 2% FBS control medium was 12 hours, which extended to 14 hours in the presence of 1 μM CGP42112A, while in 0.1% FBS control the MoCR doubling time was approximately 30 hours and this extended to 38 hours with 1 μM CGP42112A treatment. However, the effects of treatment were most easily seen by examining the number of cells in each division at the different time points. At each time point different divisions were most common, with 24 hours having few divided cells, while at 120 hours the majority of cells had divided between 7 and 10 times. Data from these cell majorities are discussed.

**Figure 1:**
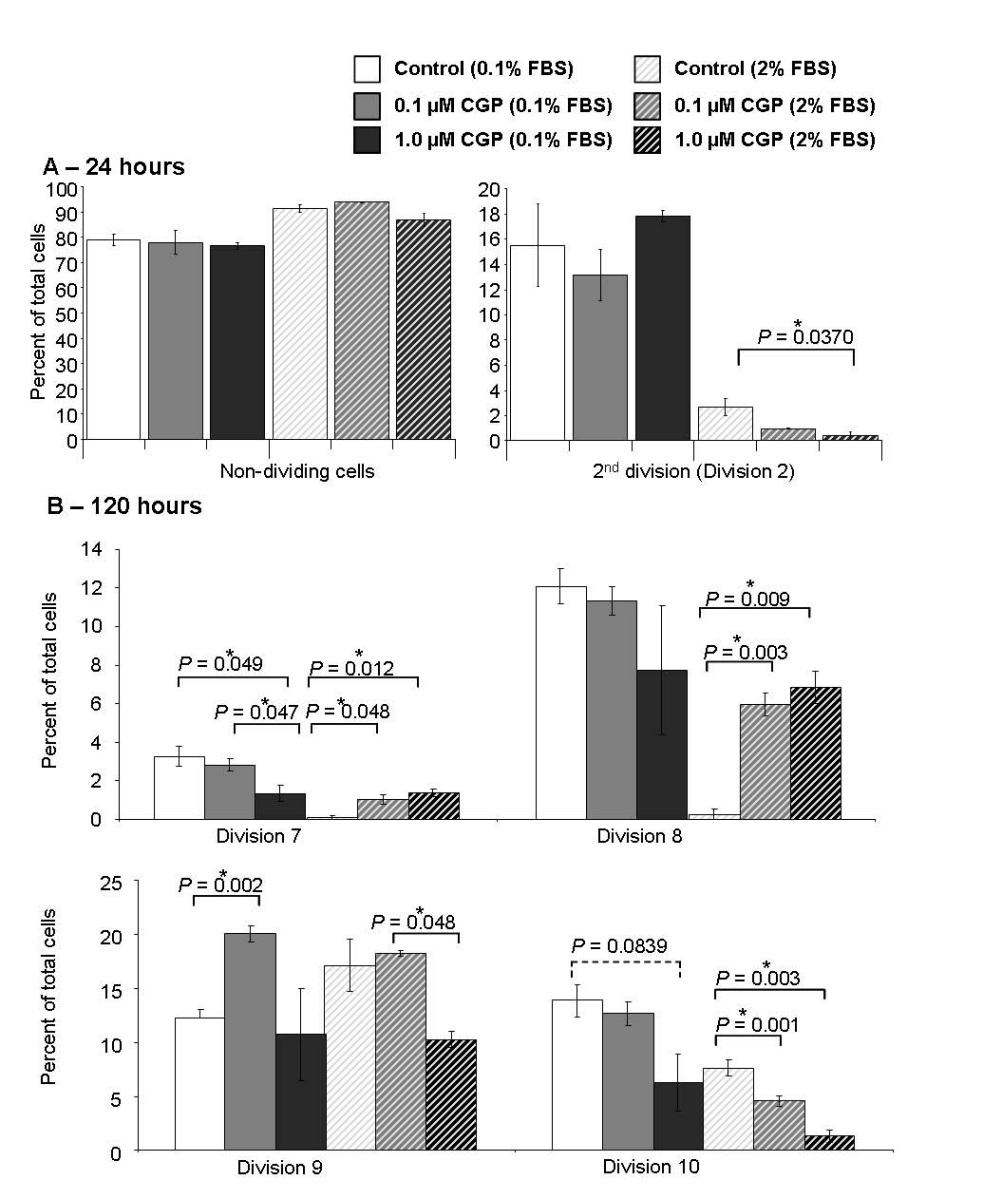
**CFSE staining of MoCR cells after 24 (A) and 120 (C) hours of treatment**. The percent of cells at each cell division was assessed by measuring the level of CFSE fluorescence by FACs. Only divisions with sufficient numbers of cells were assessed. At 24 hours almost all cells were either undivided or had divided twice, while by 120 hours all cells had divided at least 8 times. Proliferation was, in general, inhibited by CGP42112A treatment as indicated by the lower percentage of cells with more divisions. Reduced divisions were also seen at divisions 5 to 7 at time points 48 and 72 hours (*P *values between 0.058 and 0.088). Significant P values are shown with an * and solid line, while those of interest with P values between 0.1 and 0.05 are indicated with a dotted line and no *. Data are presented as mean ± S.E.M.

No significant difference was found in the percent of non-dividing cells between treatments (either in a background of 0.1% FBS or 2% FBS) after 24 hours of treatment (Figure 1A). However, the percentage of cells that divided twice over 24 hours was significantly reduced by 1 μM CGP42112A treatment compared to 2% FBS control (*P *= 0.0370, t-test). This was not the case when cells were cultured in a 0.1% FBS background. Similar reductions (although failing to reach significance, *P *between 0.058 and 0.088) were seen for 48 and 72 hour treatments (data not shown). After 120 hours, proliferation in both the 0.1% FBS and 2% FBS background was inhibited by CGP42112A treatment with the higher concentration having a greater inhibitory effect. This was evident by the increase in the percent of treated cells in the 7^th ^and 8^th ^divisions (*P *≤ 0.0479) in the 2% FBS background compared to the percent in the later 9 ^th ^and 10 ^th ^divisions, in which there were more control than treated cells and significantly so for the 10^th ^division (*P *≤ 0.0030) (Figure 1B). In the 0.1% FBS background all divisions between 7 and 10, with the 7^th ^significantly reduced (*P *= 0.049), by 1 μM CGP42112A treatment.

### CGP42112A promoted apoptosis of MOCR cells in vitro

**Figure 2:**
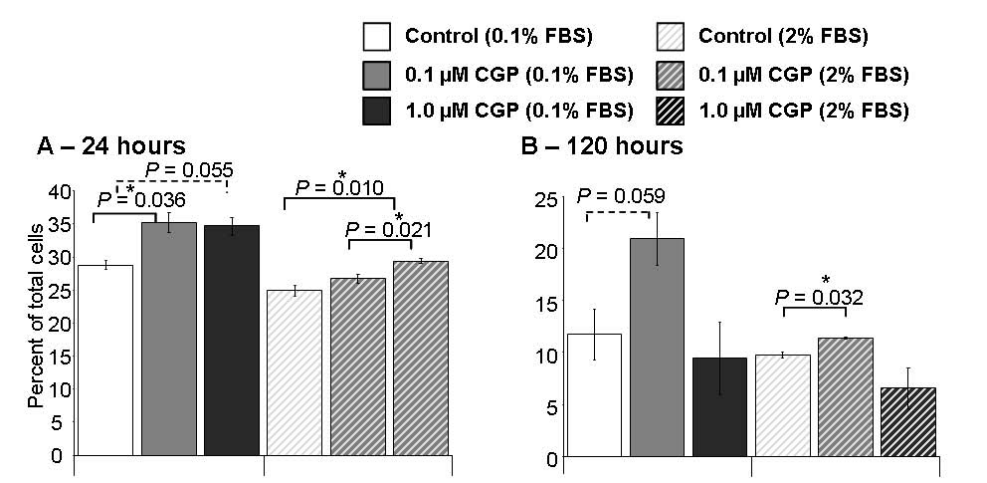
**Percent PI-positive MoCR cells**. The percent of PI-positive (apoptotic) cells after 24 and 120 hours was assessed using FACs at the same time as CFSE analysis. Although the concentrations of CGP-421112A had their most significant effects at different times and under different background conditions, in general, CGP42112A treatment increased apoptosis. Significant P values are shown with an * and solid line, while those of interest with P values between 0.1 and 0.05 are indicated with a dotted line and no *. Data are presented as mean ± S.E.M.

1 μM CGP42112A treatment for 24 hours significantly increased the percent of apoptotic MoCR cells when cultured in a background of 2% FBS compared to control (*P *= 0.0097, t-test; Figure 2A). Similar increases in apoptosis, although failing to reach significance, were seen at 48 and 72 hours. In the 0.1% FBS background, apoptosis was increased by both 0.1 and 1 μM CGP42112A, reaching significance for the 0.1 μM treatment (*P *= 0.0361). After 120 hours of treatment (Figure 2B), this increase in apoptosis remained evident and was significant in the 2% FBS background (*P *= 0.0323), while in the 0.1% FBS background the data was inconclusive but suggestive of a similar trend (*P *= 0.0595).

### CGP42112A treatment increased iNOS and PLA2 staining in vitro

**Figure 3:**
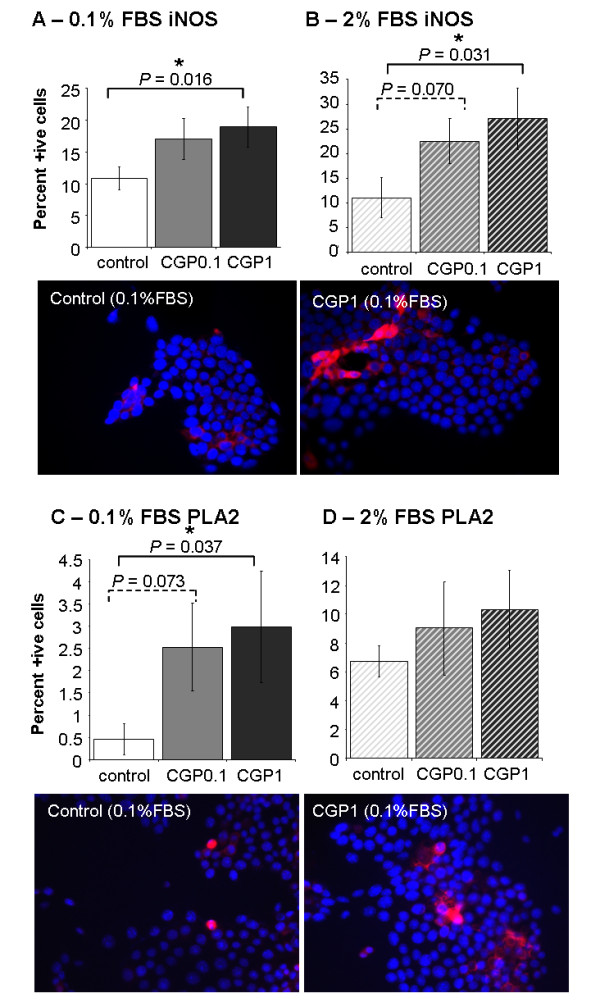
**iNOS and PLA2 staining of MoCR cells cultured for 24 hours with CGP42112A at 0.1 or 1 μM in a background of either 0.1% FBS or 2% FBS**. Representative images are shown below. Both iNOS and PLA2 staining was increased by CGP42112A treatment. Significant P values are shown with an * and solid line, while those of interest with P values between 0.1 and 0.05 are indicated with a dotted line and no *. Data are presented as mean ± S.E.M.

The number of iNOS positive cells was significantly increased by 24 hours treatment with 1 μM CGP42112A (*P *= 0.0156) and while 0.1 μM CGP42112A treatment also increased the percent positive MoCR cells compared to control this was not significant (Figure 3A). Similarly, in the 2% FBS background, 1 μM treatment with CGP42112A increased iNOS staining (*P = *0.0311). PLA2, a key signalling molecule induced by AT2R activation [18,19], showed a similar result with increased staining corresponding to increasing concentrations of CGP42112A treatment (Figure 3B). This increase in PLA2 was consistent between 2% and 0.1% FBS backgrounds, but reached significance only in the 0.1% FBS background (*P *= 0.0374)

### CGP42112A treatment decreased tumour burden in vivo

**Figure 4:**
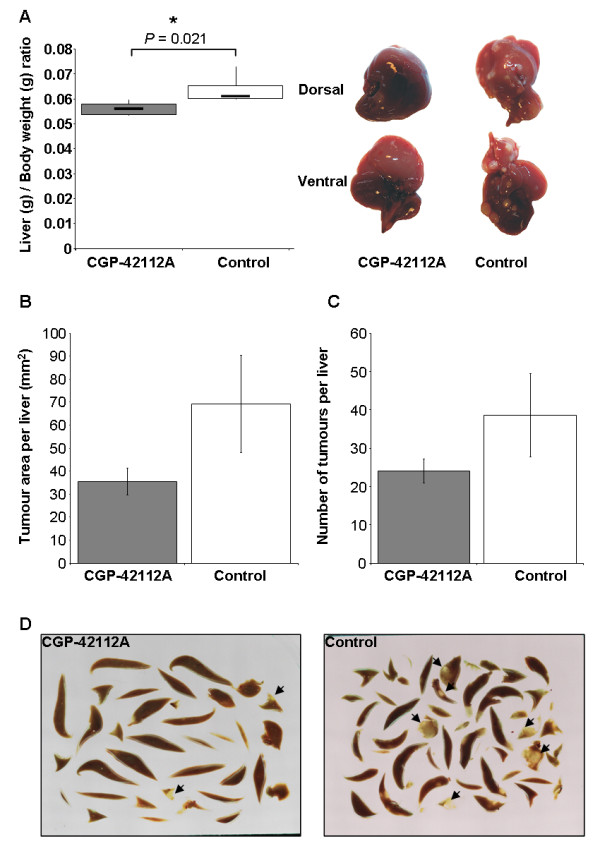
**Liver to body weight ratio for CGP42112A treated and untreated mice (A)**. Representative images are also presented. CGP42112A treatment significantly decreased the liver to body weight ration, indicating reduced tumour burden in these animals compared to control. The mean area of tumour per sectioned liver (B) and the number of tumours per liver (C) also showed reductions with CGP42112A treatment compared to control, although these did not reach significance. Images of fractionated liver sections sued to count the number of tumours and tumour area are also shown, examples of tumours are indicated by an arrow head (D). Significant P values are shown with an * and solid line, while those of interest with P values between 0.1 and 0.05 are indicated with a dotted line and no *. Data are presented as mean ± S.E.M.

A syngenic orthotopic model of CRC liver metastases was used to assess the potential of AT2R activation (via CGP42112A) to inhibit tumour growth. Mice were induced with CRC liver metastases and treated CGP42112A until termination of the experiment at day 21. Animals in the control group received no treatment. The liver to body weight ratio was used as an indicator of liver tumour burden. CGP42112A treatment resulted in a significant decrease in tumour load (liver to body weight ratio) (*P *= 0.021, Bonferroni t-test) (Figure 4A). There was no significant difference in body weight between the control and CGP42112A-treated animals, suggesting that could account for the decrease in liver to body weight ratio. We also found that the average area of all tumours in the liver for the control group was higher than that of the treatment group (Figure 4B) and that the number of tumours per liver was decreased by CGP42112A treatment (Figure 4C). However, neither of these differences reached significance.

### CGP42112A decreased proliferation and apoptosis of tumour cells growing in the liver

**Figure 5:**
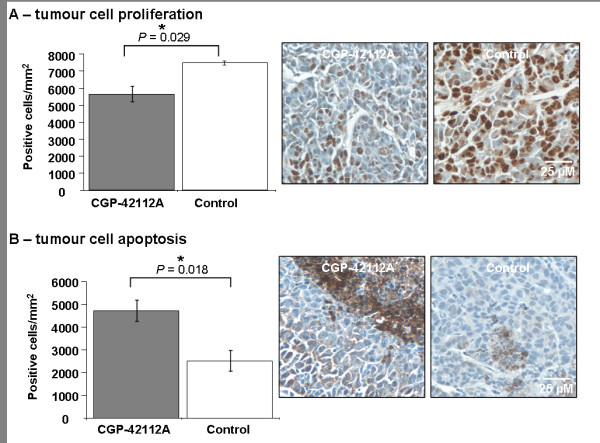
**PCNA staining of CRC cells growing in the liver (A) shows that CGP42112A treatment inhibited MoCR proliferation *in vivo***. Immunostaining for active caspase 3 also confirmed a treatment-induced increase in cancer cell apoptosis (B) in the same *in vivo *model. MoCR metastases were induced in the liver of CBA mice and allowed to grow for 21 days before fixing in PFA and immunohistochemcial analyses. Significant P values are shown with an * and solid line, while those of interest with P values between 0.1 and 0.05 are indicated with a dotted line and no *. Data are presented as mean ± S.E.M.

Immunohistochemical staining for PCNA on tumour bearing liver specimens was performed to determine if CGP42112A could inhibit proliferation of cancer cells *in vivo*. CGP42112A (*P *= 0.029, Games-Howell) treatment caused a reduction in proliferation of tumour cells (Figure 5A). Immunohistochemical staining for active Caspase-3 was used to distinguish apoptotic cancer cells for quantitative analysis. CGP42112A treatment resulted in a significant increase (*P *= 0.018, Bonferroni t-test) in apoptosis of tumour cells growing in the liver compared to controls (Figure 5B).

### CGP42112A increased secreted VGEF from MoCR cells in vitro

**Figure 6:**
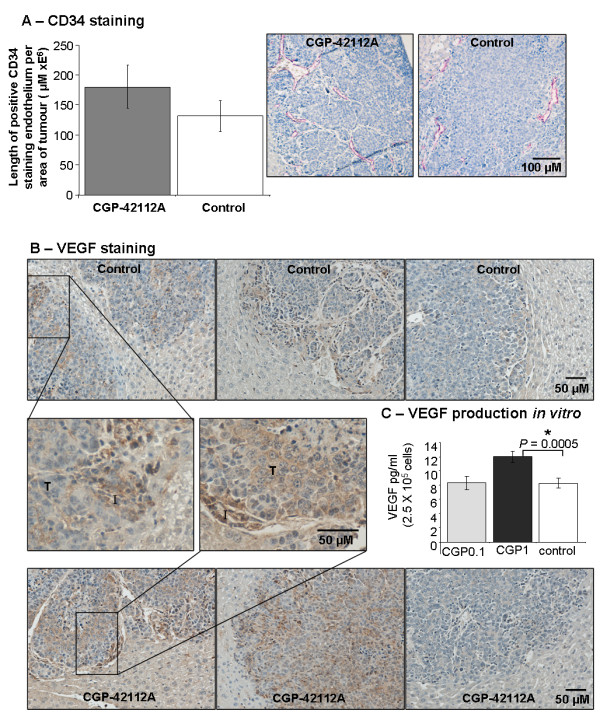
**CD34 staining of neoangiogenic vessels in treated and untreated animals inoculated with MoCR cells in the liver (A)**. Examples of high, medium, and low VEGF-expressing tumours (MoCR metastases growing in the liver) are shown for both control and treated animals (from left to right, respectively). Tumour (T) and infiltrating (I) cells are indicated. ELISA was used to examine VEGF secreted into medium conditioned by MoCR cells treated with CGP42112A at either 1 μM or 0.1 μM in a background of 0.1% FBS/RPMI. Significant P values are shown with an * and solid line, while those of interest with P values between 0.1 and 0.05 are indicated with a dotted line and no *. Data are presented as mean ± S.E.M.

CGP42112A treatment was also associated with an increase in CD34 staining *in vivo *(Figure 6A). While, this increase failed to reach significance, evidence for a pro-angiogenic role of AT2R activation is indicated by the increase in VEGF secreted by MoCR cells both *in vivo *(Figure 6B) and *in vitro *(Figure 6C). *In vivo*, VEGF staining in tumours and in tumour infiltrating cells was highly variable in control and treated animals as well as between tumours within animals. However, in highly stained tumours treated animals only showed significant tumour cell-associated VEGF, as opposed to the tumour infiltrating cells. Figure 6B inset (higher magnification) shows VEGF-positive tumour and infiltrating cells in a tumour from a CGP42112A treated animal, while infiltrating cells were positive in the control. Supporting this observation, *in vitro *1 μM CGP42112A treatment increased the level of VEGF secreted into culture medium by MoCR cells after 24 hours (*P *= 0.0005, t-test) (Figure 6C).

### CGP42112A treatment was not associated with any obvious renal or pleural abnormalities

**Figure 7:**
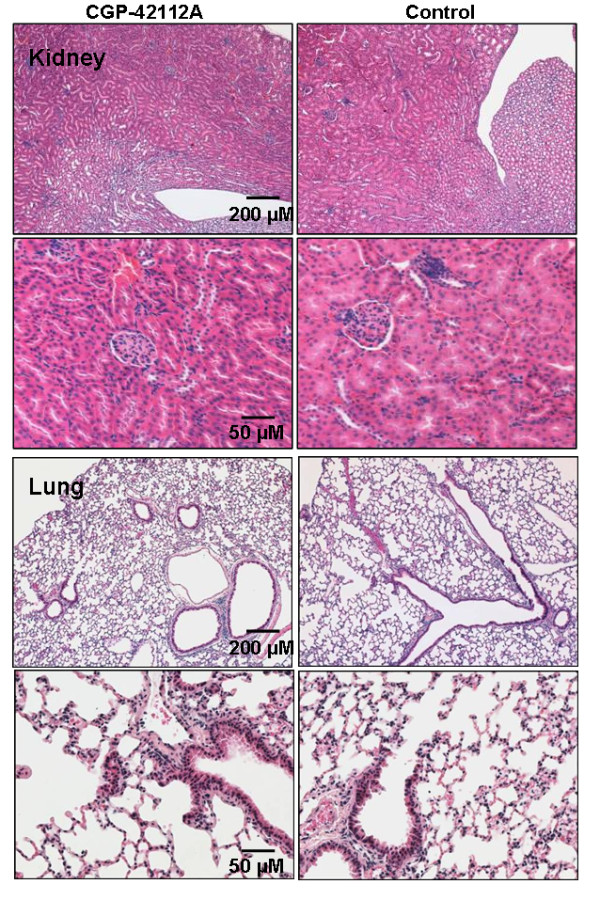
**Representative images of the kidney (upper panels) and lung (lower panel) for treated and control mice**. No histological differences could be discerned.

Lung and kidney tissues were collected to ensure that these organs, both of which are important in the systemic RAS and which have important local RAS, were unaltered by treatment (Figure 7). This analysis was important as, to the best of the authors knowledge, CGP42112A has not yet been tested *in vivo *for the length of time studied here. No notable changes in either the lungs or kidneys between treated and control groups were found nor was there any evidence of altered behaviour or general condition between control and treatment groups.

## Discussion

The RAS is now known to contribute to the regulation of tumour growth in several types of malignancy, but to date most research has focused on the inhibitory potential of blocking the classical RAS pathway, namely AT1R blockade or ACE inhibition [20-23]. A few studies have investigated the effect of targeting the MasR, via infusion of its ligand ANG-(1-7)[24-26], but no studies have examined the potential of AT2R activation in an anti-cancer setting. Therefore, it was the aim of this study to establish the potential of targeting the AT2R to inhibit tumour growth in a model of CRC liver metastases.

AT2R activation by the AT2R agonist (CGP42112A) significantly reduced proliferation and increased apoptosis of MoCR cells *in vitro*. Supporting these results, activation of the AT2R with 0.6 μg/kg/hr of CGP42112A also induced apoptosis and decreased proliferation of MoCR cells growing in the mouse liver. Our results are supported by those of others demonstrating a AT2R mediated increase in apoptosis of a rat pheochromocytoma cell line (PC12W) *in vitro*[27] and endothelial cells in an *in vivo *ischemia induced angiogenesis model[10], while AT2R activation in rat coronary endothelial cells and vascular smooth muscle cells inhibited proliferation [11].

Regulation of tumour angiogenesis is a key mechanism by which the AT1R regulates tumour growth [20,21,23,28]. ANG II activation of the AT1R is associated with VEGF secretion and tumour angiogenesis is suppressed following AT1R blockade [23,29]. Both the AT1R and AT2R stimulate VEGF secretion by rat pituitary tumour cells [13] and the AT2R is highly expressed in intratumoural blood vessels of human pituitary adenomas[12]. However, the AT2R can also inhibit VEGF signalling [9] and angiogenesis [10]. Therefore, the role of the AT2R in angiogenesis appears to be plastic; although what determines whether the AT2R mediates pro- or anti-angiogenic actions is not known. Here we found a slight increase in the level of CD34 positive staining endothelium in the tumours of CGP42112A treated mice and a significant increase in VEGF secreted by CGP42112A treated MoCR cells *in vitro*. The lack of a significant effect *in vivo *may reflect the low dose of CGP42112A used or a contribution by the tumour microenvironment. However, it also appeared that VEGF, while primarily derived from tumour-infiltrating cells in both treated and untreated animals, was produced to a far greater extent by cancer cells in treated animals. Interestingly, Clere et al. (2010) similarly found that in fibrosarcoma the AT2R could promote VEGF production and, in this case, promote tumourigenesis also through increased cell proliferation [30].

The AT2R can directly or indirectly, via stimulation of bradykinin receptor, induce nitric oxide (NO) production [31]. We found that CGP42112A treatment for 24 hours significantly increased the number of iNOS positive MoCR cells *in vitro*. Activation of the AT2R (either by endogenous ligand or CGP42112A) has been shown to increase eNOS levels in the developing pig [32] and nNOS in the rat kidney[33]. Moreover, inhibition of iNOS by NG-monomethyl-L-arginine mimicked AT2R inhibition in aortic vasodilation[34]. NO can either promote or inhibit tumour progression depending on the localisation of NOS isoforms, concentration and duration of NO exposure, and cellular sensitivity to NO [35]. Thus, the consequences of the iNOS upregulation are likely to be complex, especially if stromal or host cells are also responsive to CGP42112A treatment. However, the increase in NO could contribute to both the increase in neovascularisation and apoptosis described *in vivo*.

The increase in PLA2 was not as evident as that of iNOS (possibly due to differences in the quality of the antibody, as a greater standard error was seen for PLA2 stained cells). However, PLA2 is a key player in the generation of arachidonic acid and, subsequently, prostaglandins which are known to stimulate angiogenesis, in part through an increase in NO production [36]. Therefore, it is possible that the increase in iNOS described above reflects increased PLA2 activation by the AT2R.

Despite the pro-angiogenic effects of CGP42112A, and perhaps as a consequence of its apoptotic and anti-proliferative effects, AT2R activation resulted in a significant reduction in the liver to body weight ratio, indicating a reduced tumour burden in the liver. There was also a reduction in the number of tumours and in the average area of tumours, although these effects were not significant. These results were observed despite the relatively low dose of CGP42112A used (0.6 μg/kg/hr). This dose was chosen because CGP42112A had not been used for the length of time examined here (21 days) nor had it been tested in the context of a cancer model and so the potential for side effects were unknown. However, histological examination of the kidneys and lungs (as well as the normal liver surrounding tumours) failed to find any evidence of a deleterious effects relating to CGP42112A treatment. In rats, doses of 6000 μg/kg/hr have been used for up to 14 days [14], thus there is considerable scope for higher doses to be tested in our model.

Given the promising initial results demonstrating a reduction in MoCR proliferation and induction of apoptosis, reduced tumour burden, and a lack of side-effects, additional studies confirming the potential of targeting the AT2R in cancer are not warranted. AT2R expression is low in most adult tissues [37], but is frequently up-regulated in patients with gastric cancer [38]. Thus, targeting this receptor might have few side-effects. However, given the positive correlation between tumour-angiogenesis and poor patient outcomes, the possible increase in angiogenesis resulting from CGP42112A treatment must be examined further. Nevertheless, the angiogenic functions of the AT2R are known to be variable and may lessen even with higher doses of CGP42112A. Alternatively, if AT2R activation does indeed increase angiogenesis this effect could be counteracted by a combination with anti-angiogenic therapies.

## Conclusions

The results presented here demonstrate the potential of the AT2R as a target for inhibiting growth of CRC liver metastases by decreasing cancer cell proliferation and apoptosis. However, a caveat to its use would be its possible pro-angiogenic effects. Given the grim prospects for patients diagnosed with metastatic CRC, the potential of novel targets for improving patient outcomes is of great significance.

## Abbreviations

ANG II: angiotensin II; ANG-(1-7): angiotensin-(1-7); ACE: angiotensin converting enzyme; AT1R: angiotensin II type 1 receptor; AT2R: angiotensin II type 2 receptor; CRC: colorectal cancer; MasR: mitochondrial assembly receptor; NO: nitric oxide; iNOS: inducible nitric oxide synthase; PLA2: phospholypase A2; RAS: renin angiotensin system; VEGF: vascular endothelial growth factor.

## Competing interests

The authors declare that they have no competing interests.

## Authors' contributions

EA conceived of the study, coordinated the research, performed and analysed CD34, VEGF, PLA2, and iNOS staining, VEGF ELISA, CFSE and PI staining, and wrote the manuscript. WC carried out PCNA and caspase 3 immunohistochemical studies, analysed tumour burden, and performed the statistical analysis. SW participated in animal studies. CC contributed to the design and concept of the study. All authors reviewed the manuscript.

## References

[B1] McLoughlin JM Jensen EH Malafa M Resection of colorectal liver metastases: current perspectives Cancer Control 2006 13 1 32 41 16508624 10.1177/107327480601300105

[B2] Lau WY Lai EC Hepatic resection for colorectal liver metastases Singapore Med J 2007 48 7 635 639 17609825

[B3] Stangl R Altendorf-Hofmann A Charnley RM Scheele J Factors influencing the natural history of colorectal liver metastases Lancet 1994 343 8910 1405 1410 10.1016/S0140-6736(94)92529-1 7515134

[B4] Deshayes F Nahmias C Angiotensin receptors: a new role in cancer? Trends Endocrinol Metab 2005 16 7 293 299 10.1016/j.tem.2005.07.009 16061390

[B5] Ager EI Neo J Christophi C The renin-angiotensin system and malignancy Carcinogenesis 2008 29 9 1675 1684 10.1093/carcin/bgn171 18632755

[B6] Uemura H Ishiguro H Nakaigawa N Nagashima Y Miyoshi Y Fujinami K Sakaguchi A Kubota Y Angiotensin II receptor blocker shows antiproliferative activity in prostate cancer cells: a possibility of tyrosine kinase inhibitor of growth factor Molecular cancer therapeutics 2003 2 11 1139 1147 14617787

[B7] Fujita M Hayashi I Yamashina S Itoman M Majima M Blockade of angiotensin AT1a receptor signaling reduces tumor growth angiogenesis, and metastasis Biochemical and biophysical research communications 2002 294 2 441 447 10.1016/S0006-291X(02)00496-5 12051731

[B8] Neo JH Malcontenti-Wilson C Muralidharan V Christophi C Effect of ACE inhibitors and angiotensin II receptor antagonists in a mouse model of colorectal cancer liver metastases J Gastroenterol Hepatol 2007 22 4 577 584 10.1111/j.1440-1746.2006.04797.x 17376054

[B9] Fujiyama S Matsubara H Nozawa Y Maruyama K Mori Y Tsutsumi Y Masaki H Uchiyama Y Koyama Y Nose A Angiotensin AT(1) and AT(2) receptors differentially regulate angiopoietin-2 and vascular endothelial growth factor expression and angiogenesis by modulating heparin binding-epidermal growth factor (EGF)-mediated EGF receptor transactivation Circulation research 2001 88 1 22 29 11139469 10.1161/01.res.88.1.22

[B10] Silvestre JS Tamarat R Senbonmatsu T Icchiki T Ebrahimian T Iglarz M Besnard S Duriez M Inagami T Levy BI Antiangiogenic effect of angiotensin II type 2 receptor in ischemia-induced angiogenesis in mice hindlimb Circulation research 2002 90 10 1072 1079 10.1161/01.RES.0000019892.41157.24 12039796

[B11] Stoll M Steckelings UM Paul M Bottari SP Metzger R Unger T The angiotensin AT2-receptor mediates inhibition of cell proliferation in coronary endothelial cells The Journal of clinical investigation 1995 95 2 651 657 10.1172/JCI117710 7860748 PMC295531

[B12] Pawlikowski M Immunohistochemical detection of angiotensin receptors AT1 and AT2 in normal rat pituitary gland, estrogen-induced rat pituitary tumor and human pituitary adenomas Folia histochemica et cytobiologica/Polish Academy of Sciences Polish Histochemical and Cytochemical Society 2006 44 3 173 177 16977796

[B13] Ptasinska-Wnuk D Lawnicka H Fryczak J Kunert-Radek J Pawlikowski M Angiotensin peptides regulate angiogenic activity in rat anterior pituitary tumour cell cultures Endokrynologia Polska 2007 58 6 478 486 18205103

[B14] Hirano T Ran J Adachi M Opposing actions of angiotensin II type 1 and 2 receptors on plasma cholesterol levels in rats Journal of hypertension 2006 24 1 103 108 10.1097/01.hjh.0000198030.30095.98 16331107

[B15] Suzuki K Han GD Miyauchi N Hashimoto T Nakatsue T Fujioka Y Koike H Shimizu F Kawachi H Angiotensin II type 1 and type 2 receptors play opposite roles in regulating the barrier function of kidney glomerular capillary wall The American journal of pathology 2007 170 6 1841 1853 10.2353/ajpath.2007.060484 17525253 PMC1899458

[B16] Kuruppu D Christophi C Bertram JF O'Brien PE Characterization of an animal model of hepatic metastasis J Gastroenterol Hepatol 1996 11 1 26 32 10.1111/j.1440-1746.1996.tb00006.x 8672738

[B17] Ran J Hirano T Fukui T Saito K Kageyama H Okada K Adachi M Angiotensin II infusion decreases plasma adiponectin level via its type 1 receptor in rats: an implication for hypertension-related insulin resistance Metabolism: clinical and experimental 2006 55 4 478 488 16546478 10.1016/j.metabol.2005.10.009

[B18] Lemarie CA Schiffrin EL The angiotensin II type 2 receptor in cardiovascular disease J Renin Angiotensin Aldosterone Syst 11 1 19 31 10.1177/1470320309347785 19861349

[B19] Shi ST Li YF Interaction of signal transduction between angiotensin AT1 and AT2 receptor subtypes in rat senescent heart Chinese medical journal 2007 120 20 1820 1824 18028779

[B20] Yoshiji H Kuriyama S Kawata M Yoshii J Ikenaka Y Noguchi R Nakatani T Tsujinoue H Fukui H The Angiotensin-I-converting Enzyme Inhibitor Perindopril Suppresses Tumor Growth and Angiogenesis: Possible Role of the Vascular Endothelial Growth Factor Clinical Cancer Research 2001 7 1073 1078 11309359

[B21] Kosaka T Miyajima A Takayama E Kikuchi E Nakashima J Ohigashi T Asano T Sakamoto M Okita H Murai M Angiotensin II type 1 receptor antagonist as an angiogenic inhibitor in prostate cancer Prostate 2007 67 1 41 49 10.1002/pros.20486 17044086

[B22] Kowalski J Belowski D Madej A Herman ZS Effects of thiorphan bestatin and captopril on the Lewis lung carcinoma metastases in mice Pol J Pharmacol 1995 47 5 423 427 8868134

[B23] Suganuma T Ino K Shibata K Kajiyama H Nagasaka T Mizutani S Kikkawa F Functional expression of the angiotensin II type 1 receptor in human ovarian carcinoma cells and its blockade therapy resulting in suppression of tumor invasion angiogenesis, and peritoneal dissemination Clin Cancer Res 2005 11 7 2686 2694 10.1158/1078-0432.CCR-04-1946 15814650

[B24] Gallagher PE Tallant EA Inhibition of human lung cancer cell growth by angiotensin-(1-7) Carcinogenesis 2004 25 11 2045 2052 10.1093/carcin/bgh236 15284177

[B25] Menon J Soto-Pantoja DR Callahan MF Cline JM Ferrario CM Tallant EA Gallagher PE Angiotensin-(1-7) inhibits growth of human lung adenocarcinoma xenografts in nude mice through a reduction in cyclooxygenase-2 Cancer research 2007 67 6 2809 2815 10.1158/0008-5472.CAN-06-3614 17363603

[B26] Soto-Pantoja DR Menon J Gallagher PE Tallant EA Angiotensin-(1-7) inhibits tumor angiogenesis in human lung cancer xenografts with a reduction in vascular endothelial growth factor Molecular cancer therapeutics 2009 8 6 1676 1683 10.1158/1535-7163.MCT-09-0161 19509262 PMC3314264

[B27] Horiuchi M Hayashida W Kambe T Yamada T Dzau VJ Angiotensin type 2 receptor dephosphorylates Bcl-2 by activating mitogen-activated protein kinase phosphatase-1 and induces apoptosis The Journal of biological chemistry 1997 272 30 19022 19026 10.1074/jbc.272.30.19022 9228085

[B28] Egami K Murohara T Shimada T Sasaki K Shintani S Sugaya T Ishii M Akagi T Ikeda H Matsuishi T Role of host angiotensin II type 1 receptor in tumor angiogenesis and growth The Journal of clinical investigation 2003 112 1 67 75 12840060 10.1172/JCI16645 PMC162282

[B29] Kosugi M Miyajima A Kikuchi E Horiguchi Y Murai M Angiotensin II type 1 receptor antagonist candesartan as an angiogenic inhibitor in a xenograft model of bladder cancer Clin Cancer Res 2006 12 9 2888 2893 10.1158/1078-0432.CCR-05-2213 16675585

[B30] Clere N Corre I Faure S Guihot AL Vessieres E Chalopin M Morel A Coqueret O Hein L Delneste Y Deficiency or blockade of angiotensin II type 2 receptor delays tumorigenesis by inhibiting malignant cell proliferation and angiogenesis International journal of cancer 2010 in press 10.1002/ijc.25234 20143398

[B31] Abadir PM Carey RM Siragy HM Angiotensin AT2 receptors directly stimulate renal nitric oxide in bradykinin B2-receptor-null mice Hypertension 2003 42 4 600 604 10.1161/01.HYP.0000090323.58122.5C 12953015

[B32] Ratliff B Sekulic M Rodebaugh J Solhaug MJ Angiotensin II regulates nitric oxide synthase expression in afferent arterioles of the developing porcine kidney Pediatric research 2010 68 1 29 34 20386492 10.1203/PDR.0b013e3181e12770 PMC2891964

[B33] Siragy HM Carey RM The subtype 2 (AT2) angiotensin receptor mediates renal production of nitric oxide in conscious rats The Journal of clinical investigation 1997 100 2 264 269 10.1172/JCI119531 9218502 PMC508188

[B34] Lee JH Xia S Ragolia L Upregulation of AT2 receptor and iNOS impairs angiotensin II-induced contraction without endothelium influence in young normotensive diabetic rats Am J Physiol Regul Integr Comp Physiol 2008 295 1 R144 154 18463192 10.1152/ajpregu.00191.2008 PMC2494818

[B35] Fukumura D Kashiwagi S Jain RK The role of nitric oxide in tumour progression Nat Rev Cancer 2006 6 7 521 534 10.1038/nrc1910 16794635

[B36] Namkoong S Lee SJ Kim CK Kim YM Chung HT Lee H Han JA Ha KS Kwon YG Kim YM Prostaglandin E2 stimulates angiogenesis by activating the nitric oxide/cGMP pathway in human umbilical vein endothelial cells Exp Mol Med 2005 37 6 588 600 16391520 10.1038/emm.2005.72

[B37] Gallinat S Busche S Raizada MK Sumners C The angiotensin II type 2 receptor: an enigma with multiple variations Am J Physiol Endocrinol Metab 2000 278 3 E357 374 10710489 10.1152/ajpendo.2000.278.3.E357

[B38] Rocken C Rohl FW Diebler E Lendeckel U Pross M Carl-McGrath S Ebert MP The angiotensin II/angiotensin II receptor system correlates with nodal spread in intestinal type gastric cancer Cancer Epidemiol Biomarkers Prev 2007 16 6 1206 1212 10.1158/1055-9965.EPI-05-0934 17548686

