# Correction: Targeting the angiotensin II type 2 receptor (AT2R) in colorectal liver metastases

**DOI:** 10.1186/1475-2867-10-37

**Published:** 2010-10-21

**Authors:** Eleanor I Ager, Way W Chong, Shu-wen Wen, Christopher Christophi

**Affiliations:** 1 Department of Surgery, The University of Melbourne, Austin Health, Heidelberg, Victoria, Australia

## Correction

After publication of this work [1], we noted that we inadvertently failed to properly acknowledge all of our funding agencies. The acknowledgements section has now been modified accordingly.
